# Reliability of an Integrated Inertial Sensor for the Continuous Measurement of Active Cervical Range of Motion in a Group of Younger and Elderly Individuals

**DOI:** 10.3390/jfmk5030058

**Published:** 2020-08-03

**Authors:** Stefano Gobbo, Barbara Vendramin, Enrico Roma, Federica Duregon, Danilo Sales Bocalini, Roberta Luksevicius Rica, Andrea Di Blasio, Lucia Cugusi, Manuele Bergamo, David Cruz-Díaz, Cristine Lima Alberton, Valentina Bullo, Andrea Ermolao, Marco Bergamin

**Affiliations:** 1 Sport and Exercise Medicine Division, Department of Medicine, University of Padova, Via Giustiniani, 2-35128 Padova, Italy; stefano.gobbo@unipd.it (S.G.); barbara.vendramin@unipd.it (B.V.); romaenrico94@gmail.com (E.R.); federica.duregon@unipd.it (F.D.); manuele.bergamo@gymhub.it (M.B.); valentina.bullo@unipd.it (V.B.); andrea.ermolao@unipd.it (A.E.); 2 Laboratorio de Fisiologia e Bioquimica Experimental, Centro de Educacao Fisica e Deportos, Universidade Federal do Espirito Santo (UFES), Vitoria, ES, Rua Vergueiro, 235, Liberdade, Sao Paulo SP 01504-00, Brazil; bocaliniht@hotmail.com; 3 Departamento de Educacao Fisica e Ciencias do Envelhecimento, Laboratorio de Percepcao Corporal e Movimento, Universidade Sao Judas Tadeu, Sao Paulo SP 03166-000, Brazil; robertarica@hotmail.com; 4 Department of Medicine and Sciences of Aging, G. d’Annunzio University of Chieti-Pescara, 66013 Chieti, Italy; andiblasio@gmail.com; 5 Department of Biomedical Sciences, University of Sassari, 43/B 07100 (SS) Viale San Pietro, Italy; lucia.cugusi@uniss.it; 6 Department of Health Sciences, Faculty of Health Sciences, University of Jaén, E-23071 Jaén, Spain; dcruz@ujaen.es; 7 Physical Education School, Federal University of Pelotas (Brazil) Rua Luís de Camões, Pelotas 625-96055630, Rio Grande do Sul, Brazil; tinialberton@yahoo.com.br

**Keywords:** exercise test, neck, inertial measurement unit, aging, physical fitness, reliability study, range of motion

## Abstract

The aim of this study was to evaluate the test–retest reliability of an integrated inertial sensor (IIS) for cervical range of motion assessment. An integrated inertial sensor was placed on the forehead center of thirty older adults (OA) and thirty younger adults (YA). Participants had to perform three continuous rotations, lateral bandings and flexion–extensions with their head. Test–retest reliability was assessed after 7 days. YA showed moderate to good agreement for rotation (0.54–0.82), lateral bending (0.74–0.8), and flexion–extension (0.74–0.81) movements and poor agreement for zero point (ZP). OA showed moderate to good agreement for rotation (0.65–0.86), good to excellent agreement in lateral bending (0.79–0.92), and poor to moderate agreement for flexion–extension (0.37–0.72). Zero point showed poor to moderate agreement. In conclusion, we can affirm that this IIS is a reliable device for cervical range of motion assessment in young and older adults; on the contrary, the ZP seems to be unreliable and the addition of an external reference point could help the subject to solve this shortcoming and reduce possible biases.

## 1. Introduction

In the last few years, neck pain has been defined as a frequent musculoskeletal disorder that leads to disability [1], with an annual prevalence exceeding 30% of the general population and with a prevalence of around 65% in older adults [2]. The most common and widespread causes of neck pain are injuries, work-related musculoskeletal disorders, inflammatory conditions, and rheumatic disorders. Often, neck ache is associated with a limitation of cervical movement and could generate other disturbances such as dizziness, unsteadiness, postural instability [3], and cervical proprioception impairment [4]. Moreover, nearly 50% of cases of neck pain did not resolve with or without treatment and individuals experience some degree of pain or frequent occurrences throughout their life [5]. Therefore, the needs to assess objectively the cervical range of motion (CROM) in order to select an appropriate therapeutic intervention and to monitor patients’ prognosis is gaining increasing interest [6].

Cervical movements of flexion, extension, rotation, and lateral bending can be evaluated through different methods classified as statics or dynamics: X-rays and goniometers represent the most common static tools described in the literature; in addition, dynamic acquisitions were mainly obtained using inclinometers and kinematic analysis by optoelectronic scanners [7]. Goniometers and inclinometers represent cheaper and non-invasive alternatives to radiography. However, outcomes are considerably influenced by operator experience and ability. Despite these limits, some of them were validated [8]. At the same time, the kinematic analysis turned out to be a sophisticated and reliable method to analyze 3D neck mobility, but this specific method needs an appropriate and complex setting together with expensive software to obtain valid data.

Recently, assessment devices have become much more popular among healthcare providers. Inertial sensors seem to overcome the above-described limitations of CROM assessment [9]. The popularity of inertial sensors has increased because of their wearability. In fact, they are light, easy to use, cheap, and they permit the collection of a large amount of data. They include accelerometers, gyroscopes, and magnetometers that work by simultaneously providing real-time information without physical connectors. The literature has reported that these sensors are accurate and valid, although soft tissue artefacts were still present [6]. Overall, inertial sensors had several advantages compared to other devices. Firstly, they were portable and relatively cheap, allowing data gathering in various environments [10]. Secondly, researchers use them to determine absolute orientation and track movements in three dimensions; furthermore, it was possible to obtain different parameters simultaneously, such as linear accelerations, velocities, and displacements [11]. Finally, they could be potentially used in association with other devices such as electromyography and force transducers [12].

In light of these premises, the aim of this study is to investigate the reliability of an integrated inertial sensor (IIS) for the assessment of the active CROM in a group of asymptomatic elderly and young participants.

## 2. Materials and Methods 

### 2.1. Participants

Thirty older adults (15 men and 15 women, aged over 65 years old) and thirty young adults (15 men and 15 women, aged 18–28 years old) were recruited from June to July 2015, respectively, among cultural associations in Padova, Italy and the University of Padova, Italy (Table 1). The research protocol was performed in the Sport and Exercise Medicine Division at the University of Padova, Italy. Eligibility criteria included the following: (a) for the elderly group, an age over 65 years, (b) for the young group, an age between 18 to 28 years, (c) to be available for two evaluations conducted 7 days apart. Exclusion criteria were the following: (a) past history of neurologic or cervical musculoskeletal disorders, suffering from walleye, dizziness, or other visual or vestibular disturbances, which could negatively influence study results, (b) health problems or any physical limitations that could potentially affect CROM, (c) cases of cervical pain or traumatic problems. Clinical evaluation were carried out by a physician. Each participant received information on the purpose and procedures of the study and gave written informed consent before their participation. Sample size was consistent with previous investigations examining dynamometer measures in young adults and the elderly [12,13,14]. The study complied with the current laws of Italy for research on human participants and was approved by the University of Padova (Italy), review board (N°2027, in 12/01/2015).

### 2.2. Measurement Instruments

**Figure 1:**
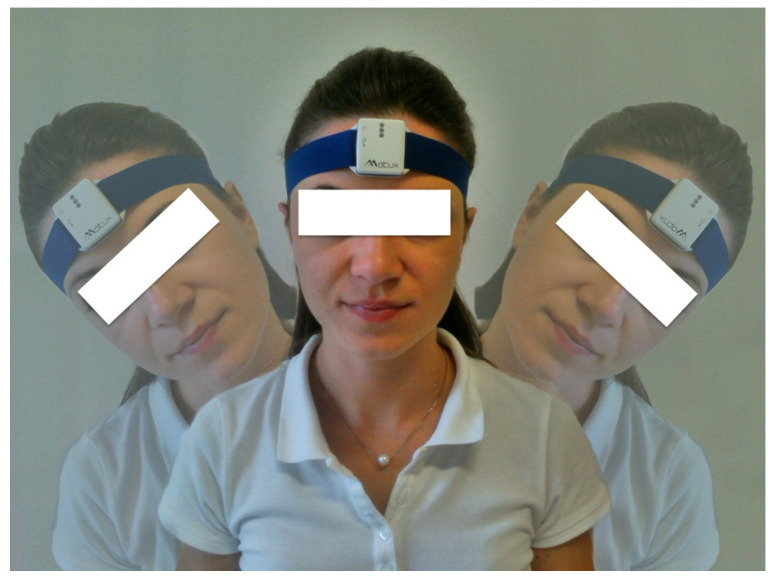
Position of the IIS and execution of lateral flexion.

Each participant was outfitted with the IIS (Motux S.r.l., Trento, Italy), placed on the forehead and tied around the head, as suggested by the manufacturer (Figure 1). The IIS was a 9-axis motion tracking device composed of a 3-axis accelerometer, 3-axis gyroscope, and 3-axis compass. Technical data of the accelerometer given by the manufacturer showed a resolution of 16 bit and a noise of 4 mg-rms and could be programmable at ±2 g, ±4 g, ±8 g, ±16 g. The acceleration sampling frequency ranges from 10 to 200 Hz. The gyroscope could be set at ±250 °/s, ±500 °/s, ±1000 °/s, ±2000 °/s. It had a resolution of 16 bit and a noise of 0.005 °/s/√Hz. The compass worked at ±1200 µT, with a resolution of 13 bit and a sensitivity of 0.3 µT /LSB. Barometer function worked from 50 to 110 kPa, with a pressure resolution of 1.5 Pa and altitude resolution of 0.3 m.

The device measured 36.4 mm × 12.5 mm (side length), 32 mm (width) without band support and 46 mm with band support. Weight was 15.8 g without band support. 

The software was integrated in the main software program and the 4Motion sensor (accelerometer, gyroscope, compass, barometer) could be used online (Bluetooth^®^ connectivity) or offline (internal logging). Wireless connection features were as follows: Bluetooth^®^ Classic v. 3.0; SPP service (serial port profile), Bluetooth^®^ profile: medical device; range (connectivity limits): 10m default—up to 60 m (optional). It was possible to use the device alone or with up to 7 sensors (depending on laptop performance).

### 2.3. Procedure 

A single inertial sensor was used in the study. It was placed on the center of the participant’s forehead, between and above the eyebrow arches. The tool was fixed around the head with a headband tight enough so that it could not fall down or create friction with hair, without causing pain. The same researcher fixed the sensor for each participant, in both trials (test–retest section). Participants were seated on a chair with an armrest and lumbar support. The neutral position with the head looking forward was considered the starting position. Participants were instructed to perform three continuous rotations on the transverse plane with their head, starting on the right. Then, they were asked to perform three continuous lateral bending movements on the coronal plane (starting on the right). Finally, they performed three continuous flexion–extension movements on the sagittal plane (starting with flexion). They were asked to execute a comfortable movement, trying to reach the maximum range of motion in every direction. Every single movement lasted 3 to 5 s each. To avoid biases by the evaluator, the same instructions were given to the participants, and no indications were provided during the execution of each trial.

Before starting the acquisition, participants were recommended to carry out each movement correctly, without combining other movements in different planes together. The software acquired data from the inertial sensor via Bluetooth^®^ connection through a computer, which was placed on a table in front of the participants. After 7 days, participants had to perform another set of measurements with the same series of movements. Participants were not restricted from exercising during the 7 days after the first trial, but they were asked not to change their usual habits. All 60 participants concluded the procedure safely, without interruptions or complications.

### 2.4. Data Post-Processing and Statistical Analysis 

Data were acquired at 50 Hz, with a gravity force of ±2 g. Data were recorded as angular degrees and were transferred from the manufactured software to Microsoft Excel 2011.

For each participant, every neck movement was analyzed, considering seven parameters:

- Zero point (ZP): the difference between the start position and the ending position;
- Maximal range of motion (ROM-max): the higher range of motion value among the three repetitions;
- Mean range of motion (ROM-med): the mean of the three ranges of motion;
- Absolute maximum excursion (Max-abs): the higher maximum excursion value from the starting position (right rotation, right lateral bending, and flexion);
- Mean maximum excursion (Max-med): the mean of the three maximum excursion values from the starting position (right rotation, right lateral bending, and flexion);
- Absolute minimum excursion (Min-abs): the higher maximum excursion value from the starting position (left rotation, left lateral bending, and extension);
- Mean minimum excursion (Min-med): the mean of the three maximum excursion values from the starting position (left rotation, left lateral bending, and extension);

The Shapiro–Wilk test was performed to test the normality of the distribution of each variable. The reliability and repeatability were examined using the limits of agreement (mean of the differences ± 1.96 × SD) derived by the Bland–Altman plot [15], while the intraclass correlation coefficient (ICC) was determined in order to measure the reliability between evaluations and trials. An ICC lower than 0.5 indicated poor reliability, values between 0.5 and 0.75 a moderate reliability, values between 0.75 and 0.9 a good reliability, and values higher than 0.9 excellent reliability [16]. The analysis was performed separately for the young and elderly groups.

## 3. Results

Thirty older adults (15 men and 15 women) and thirty young adults (15 men and 15 women) were recruited for this reliability study (Table 1).

Mean and standard deviations were calculated and reported for each parameter. The Shapiro–Wilk test indicated a normal distribution of each parameter. Test–retest mean and percentage differences for each parameter are reported in Table 2 (young sample) and Table 3 (elderly sample).

### 3.1. Test Re–Test Reliability in Young Subjects

In all three conditions, ZP showed the major test–retest mean differences in rotation (−29.27%), lateral bending (38.54%), and flexion–extension (−14.53%). Moreover, ZP showed poor agreement in each movement (rotation 0.1, lateral bending 0.29, flexion–extension 0.22).

Rotation movement showed moderate agreement of ROM-max (0.71), max-abs (0.71), min-abs (0.54), and min-med (0.66), while good agreement was found for ROM-med (0.82) and max-med (0.78).

Lateral bending movement showed good agreement for ROM-max (0.79), ROM-med (0.78), max-abs (0.8), max-med (0.77), and min-abs (0.75), while min-med reported a moderate agreement (0.74).

The flexion and extension trial showed good agreement for ROM-max (0.79), ROM-med (0.78), max-abs (0.78), max-med (0.78), and min-med (0.76), while min-abs reported moderate agreement (0.74).

### 3.2. Test Re–Test Reliability in Elderly Subjects

Similar to the young sample, the major test–retest mean differences were represented by ZP in rotation movement (−40.6%), lateral bending movement (−41.77%), and flexion–extension movement (41.59%). Moreover, ZP showed moderate agreement only for rotation movement (0.59), while lateral bending and flexion–extension showed poor agreement (0.0, 0.28).

Rotation movement showed moderate agreement for ROM-max (0.87) and ROM-med (0.84); good agreement was found for max-abs (0.7), max-med (0.7), min-abs (0.7), and min-med (0.65).

In lateral bending, the elderly group showed excellent agreement for ROM-max (0.93) and ROM-med (0.92), while max-abs (0.89), max-med (0.89), min-abs (0.8), and min-med (0.79) showed good agreement.

In terms of flexion–extension movement, the elderly group showed heterogeneous ICC values. ROM-max and ROM-med reported good agreement (0.83, 0.8), while min-abs and min-med showed moderate agreement (0.72, 0.71). On the contrary, max-abs and max-med reported poor agreement (0.42, 0.37).

## 4. Discussion

**Figure 2:**
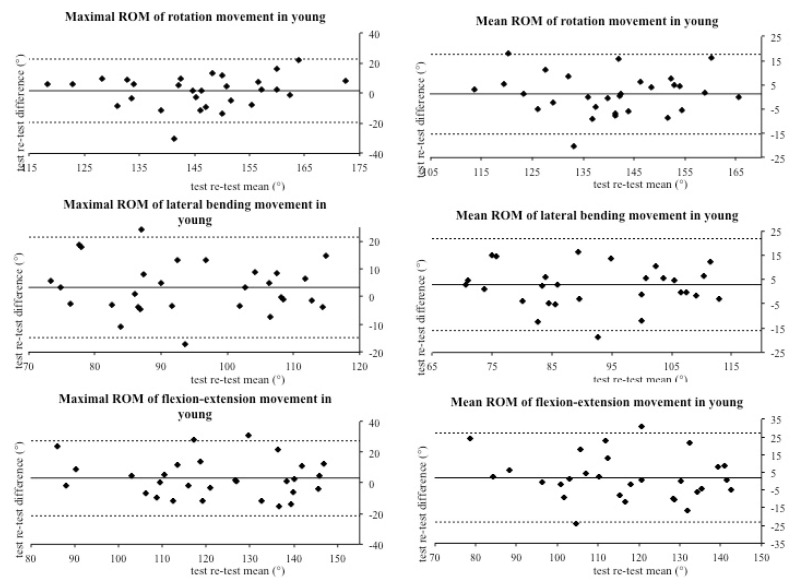
Bland–Altman plots of maximum and mean range of motion in young subjects.

**Figure 3:**
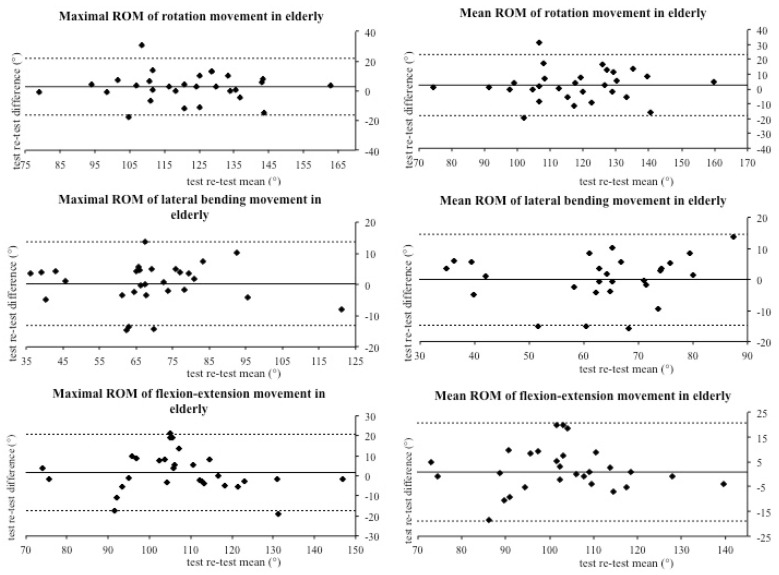
Bland–Altman plots of maximum and mean range of motion in elderly subjects.

**Figure 4:**
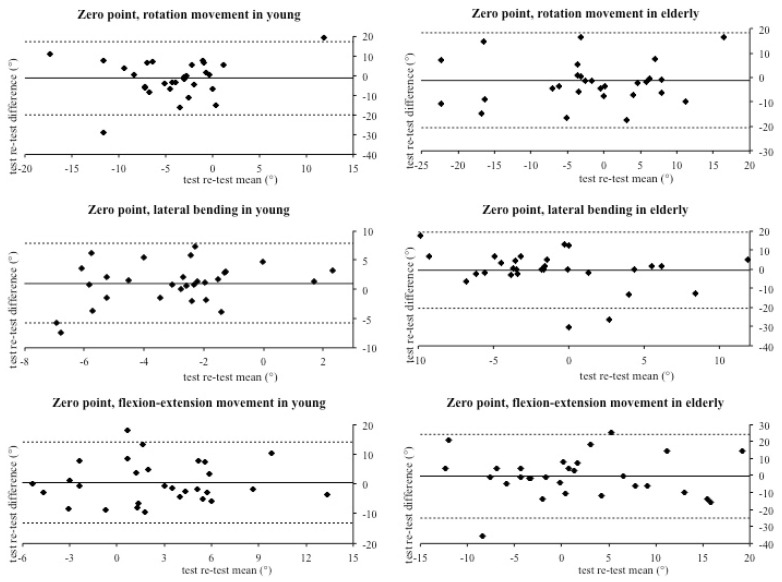
Bland–Altman plots of zero point in young and elderly subjects.

The aim of this study was to investigate the reliability of the IIS in cervical spine range of motion assessment in a group of asymptomatic subjects (young and elderly). Technological devices like the IIS have several advantages; indeed, they are small and easy to use. However, one of the most important features is the large amount and precision of data that they provide. Thus, an integrated inertial sensor with these characteristics could have several applications in many different settings, such as clinical and sport research, reducing evaluation biases. In this investigation, we compared the same cervical movements executed seven days apart, in order to quantify the repeatability of the IIS measures. Results showed moderate to good reliability, which registered overall an agreement between the analyzed values. The vast majority of values were placed within the limits of agreements and the correspondence between the test–retest difference means confirms the reproducibility of the assessments executed with the IIS. Particularly, good agreement was recorded for maximal and mean ROM for each movement in both young (Figure 2) and elderly groups (Figure 3). However, the Bland–Altman graphs (Figure 2, Figure 3 and Figure 4) showed great data variability that could be due to different participants’ habits, such as level of physical activity, job activity, and sleep quality. On the contrary, Kim and colleagues found small variability using a wireless IIS. In their protocol, they attached a sensor to the thorax to measure the compensation caused by trunk movement [9]. We hypothesize that trunk movements could affect our variability.

The most important discrepancy was noticed between the differences in the zero point. This could mean that a large number of participants did not perfectly return to the initial frontal position with their head (Figure 4). To the best of our knowledge, this start-to-end discrepancy was not observed yet and it certainly opened up a new point of discussion concerning the inability of traditional methods to quantify this effect. Different reasons could be responsible for this fact, such as vestibular causes or visuo-spatial integration components during head movements [17]. Furthermore, the head-neck movements are based on proprioception, joint positioning, and repositioning sense. Hence, the head repositioning accuracy is determined by the function of receptors located in neck joints and muscles. According to our data, Basteris and colleagues found a tendency to under or over-estimate the target position (ZP) during the head movement test [18]. 

This IIS could be adopted in different clinical settings to provide data with the aim to assess CROM and evaluate treatment effectiveness. From a non-clinical point of view, the IIS can provide real-time feedback, which could be useful for specific training and mobility assessments.

### 4.1. Limitations

This study presented several limitations. Firstly, the use of elastic bands to fix the IIS could determine minimal movement of the sensor, despite this application procedure being suggested by the manufacturer. The use of the same bands for all trials probably reduced this bias. Secondly, we advised the participants not to change their usual habits, but some parameters such as weather temperature, sleep quality, and activity of previous days could influence CROM. Thirdly, we did not perform an inter-rater comparison to assess the validity of the device but we tested its test-retest reliability. Finally, considering that the device was opportunely calibrated before its production and installation, we did not know in detail these procedures. Despite this, the bias possibility of the acquisitions was small, and the participants’ familiarization with the procedure before data collection was conducted in order to reduce the risk of bias caused by inexperience in the movements’ execution.

### 4.2. Future Investigations

Future investigations should examine more thoroughly the device applications in a clinical setting. In fact, studies that focus on cervical range analysis by inertial sensors are scarce [19]. Furthermore, although many different traditional assessment instruments are widely used, some validation studies of these tools presented several methodological inaccuracies, such as small sample size or inadequate statistical analysis [20]. Moreover, the comparison of IIS with other systems of acquisition could be an interesting way to increase the applicability of this device in different settings [21].

## 5. Conclusions

This study verified the reliability of the IIS in cervical spine range of motion assessment among young and elderly subjects. Currently, several methods on the market allow us to measure joints’ ranges of motion. Often, in clinical practice, ROM relies on goniometers, inclinometers, or—in the worst case—the naked eye, while this integrated inertial sensor represents a good alternative to traditional methods, providing more information and reducing possible manual biases. In conclusion, we can claim that this IIS is a reliable device for the assessment of cervical ROM; on the contrary, the ZP seems to be unreliable. We suggest the addition of an external device or reference point in order to reduce possible biases deriving from a return to ZP. Considering the numerous settings in which this kind of device can be employed, future research should be developed involving, for example, athletes or symptomatic patients.

## Figures and Tables

**Table 1:** Participants’ anthropometric characteristics.

N. Subjects	Weight (kg)	Height (m)	BMI (kg/m^2^)	Age (Years)
30 young (15 M, 15 F)	67.90 ± 14.66	1.74 ± 8.30	22.17 ± 3.08	22.43 ± 1.69
30 elderly (15 M, 15 F)	76.23 ± 15.79	1.70 ± 10.23	26.30 ± 3.44	68.13 ± 2.80

Abbreviation: BMI = body mass index.

**Table 2:** Results for young participants.

Variables	Test (°)	Retest (°)	Δ%	ICC (CI 95%)	Δ° (ULA-LLA 95%)
ZP Rotation	−4.84 ± 7.73	−3.42 ± 6.24	−29.27	0.1 (−0.27; 0.44)	−1.4 (17.13; −19.96)
ROM-max Rotation	146.72 ± 14.44	145.26 ± 13.05	−0.99	0.71 (0.47; 0.85)	1.46 (22.38; −19.47)
ROM-med Rotation	141.02 ± 13.69	139.83 ± 13.82	−0.84	0.82 (0.66; 0.91)	1.19 (17.65; −15.27)
Max-abs Rotation	79.23 ± 8.78	77.75 ± 7.94	−1.87	0.71 (0.47; 0.85)	1.48 (14.23; −11.27)
Max-med Rotation	75.34 ± 8.02	74.36 ± 8.21	−1.3	0.78 (0.59; 0.89)	0.98 (11.75; −9.79)
Min-abs Rotation	−68.59 ± 7.55	−68.87 ± 8.05	−0.4	0.54 (0.22; 0.75)	0.28 (15.18; −14.62)
Min-med Rotation	−65.69 ± 6.95	−65.48 ± 7.76	−0.32	0.66 (0.39; 0.83)	−0.21 (11.92; −12.34)
ZP Lat-bending	−2.54 ± 3.33	−3.52 ± 2.39	38.54	0.29 (−0.08; 0.59)	0.98 (7.79; −5.83)
ROM-max Lat-bend	96.28 ± 13.5	93.01 ± 14.3	−3.4	0.79 (0.6; 0.9)	3.27 (21.45; −14.91)
ROM-med Lat-bend	93.53 ± 13.63	90.64 ± 14.56	−3.09	0.78 (0.58; 0.89)	2.89 (21.77; −16)
Max-abs Lat-bend	49.96 ± 7.27	48.09 ± 7.32	−3.75	0.8 (0.62; 0.9)	1.87 (11.1; −7.35)
Max-med Lat-bend	48.42 ± 7.25	46.33 ± 7.35	−4.33	0.77 (0.56; 0.88)	2.10 (12.16; −7.97)
Min-abs Lat-bend	−47.04 ± 7.42	−46.06 ± 7.76	−2.07	0.75 (0.54; 0.88)	−0.98 (9.77; −11.72)
Min-med Lat-bend	−45.11 ± 7.67	−44.32 ± 8.12	−1.75	0.74 (0.52; 0.87)	−0.79 (10.57; −12.15)
ZP Flex-ext	2.77 ± 5.65	2.37 ± 5.48	−14.53	0.22 (−0.15; 0.54)	0.4 (14.05; −13.25)
ROM-max Flex-ext	123.82 ± 18.1	120.83 ± 19.35	−2.42	0.79 (0.6; 0.9)	2.99 (27.44; −21.46)
ROM-med Flex-ext	117.55 ± 17.83	115.64 ± 19.88	−1.62	0.78 (0.59; 0.89)	1.19 (26.89; −23.07)
Max-abs Flex-ext	57.79 ± 10.37	54.61 ± 10.63	−5.5	0.78 (0.58; 0.89)	3.18 (17.2; −10.84)
Max-med Flex-ext	54.22 ± 9.98	51.77 ± 11.26	−4.52	0.78 (0.58; 0.89)	2.45 (16.8; −11.89)
Min-abs Flex-ext	−67.07 ± 12.91	−67.03 ± 14.23	−0.06	0.74 (0.51; 0.87)	−0.04 (19.77; −19.84)
Min-med Flex-ext	−63.32 ± 13.21	−63.87 ± 14.19	0.86	0.76 (0.54; 0.88)	0.54 (19.77; −18.68)

Abbreviation: ULA, upper limit of agreement; LLA, lower limit of agreement; Δ%, percentage difference between test–retest values; p, p value; ICC, intraclass correlation coefficient; CI, confidence interval; Lat-bend, lateral bending; Flex-ext, flexion–extension; ZP, the difference between the start and the ending position; ROM-max, the higher ROM among the three repetitions; ROM-med, the mean of the three ROM; Max-abs, the higher maximum excursion value from the starting position (right rotation, right lateral blending and flexion); Max-med, the mean of the three maximum excursion values from the starting position (right rotation, right lateral blending, and flexion); Min-abs, the higher minimum excursion value from the starting position (left rotation, left lateral blending, and extension); Min-med, the mean of the three minimum excursion value from the starting position (left rotation, left lateral blending, and extension).

**Table 3:** Results for elderly participants.

Variables	Test (°)	Retest (°)	Δ%	ICC (CI 95%)	Δ° (ULA-LLA 95%)
ZP Rotation	−2.96 ± 10.98	−1.76 ± 10.7	−40.6	0.59 (0.3; 0.79)	−1.2 (18.31; −20.71)
ROM-max Rotation	122.25 ± 18.23	119.61 ± 18.31	−2.16	0.87 (0.74; 0.93)	2.64 (21.74; −16.47)
ROM-med Rotation	118.71 ± 18.27	116.16 ± 17.73	−2.15	0.84 (0.69; 0.92)	2.55 (23.05; −17.95)
Max-abs Rotation	68.63 ± 12.06	67.36 ± 10.99	−1.84	0.7 (0.45; 0.85)	1.27 (19.23; −16.69)
Max-med Rotation	66.31 ± 12.03	64.85 ± 10.19	−2.21	0.7 (0.45; 0.85)	1.47 (18.84; −15.91)
Min-abs Rotation	−54.58 ± 10.43	−53.6 ± 9.61	−1.79	0.7 (0.46; 0.85)	−0.98 (14.51; −16.46)
Min-med Rotation	−52.4 ± 10.31	−51.31 ± 9.52	−2.07	0.65 (0.37; 0.82)	−1.09 (15.61; −17.78)
ZP Lat-bending	−1.36 ± 6	−0.79 ± 8.16	−41.77	0 (−0.36; 0.36)	−0.57 (19.26; −20.4)
ROM-max Lat-bend	68.67 ± 17.98	68.46 ± 18.29	−0.3	0.93 (0.87; 0.97)	0.2 (13.54; −13.13)
ROM-med Lat-bend	65.51 ± 17.71	65.64 ± 18.02	0.21	0.92 (0.83; 0.96)	−0.14 (14.6; −14.87)
Max-abs Lat-bend	37.7 ± 10.23	36.47 ± 10.99	−3.27	0.89 (0.79; 0.95)	1.23 (11.14; −8.68)
Max-med Lat-bend	35.67 ± 9.88	34.33 ± 10.74	−3.78	0.89 (0.78; 0.95)	1.35 (11.12; −8.42)
Min-abs Lat-bend	−31.47 ± 9.52	−33.14 ± 9.54	5.31	0.8 (0.62; 0.9)	1.67 (13.81; −10.47)
Min-med Lat-bend	−29.83 ± 9.47	−31.32 ± 9.31	4.97	0.79 (0.61; 0.9)	1.48 (13.64; −10.68)
ZP Flex-ext	1.23 ± 10.3	1.74 ± 10.44	41.59	0.28 (−0.09; 0.58)	−0.51 (24.06; −25.09)
ROM-max Flex-ext	108.11 ± 15.48	106.43 ± 16.99	−1.56	0.83 (0.67; 0.92)	1.69 (20.73; −17.36)
ROM-med Flex-ext	103.83 ± 14.93	102.9 ± 16.17	−0.89	0.8 (0.62; 0.9)	0.92 (20.77; −18.86)
Max-abs Flex-ext	44.58 ± 9.98	47.33 ± 9.93	6.17	0.42 (0.07; 0.68)	−2.75 (18.56; −24.07)
Max-med Flex-ext	42.54 ± 10.33	45.24 ± 9.81	6.33	0.37 (0.01; 0.65)	−2.69 (19.66; −25.04)
Min-abs Flex-ext	−64.29 ± 14.31	−60.39 ± 16.83	−6.08	0.72 (0.48; 0.86)	−3.91 (19.61; −27.42)
Min-med Flex-ext	−61.28 ± 14.2	−57.66 ± 16.2	−5.9	0.71 (0.47; 0.85)	−3.62 (19.63; −26.86)

Abbreviation: ULA, upper limit of agreement; LLA, lower limit of agreement; Δ%, percentage difference between test–retest values; p, p value; ICC, intraclass correlation coefficient; CI, confidence interval; Lat-bend, lateral bending; Flex-ext, flexion–extension; ZP, the difference between the start and the ending position; ROM-max, the higher ROM among the three repetitions; ROM-med, the mean of the three ROM; Max-abs, the higher maximum excursion value from the starting position (right rotation, right lateral blending, and flexion); Max-med, the mean of the three maximum excursion values from the starting position (right rotation, right lateral blending, and flexion); Min-abs, the higher minimum excursion value from the starting position (left rotation, left lateral blending, and extension); Min-med, the mean of the three minimum excursion value from the starting position (left rotation, left lateral blending, and extension).
